# Seeds in the guts: can seed traits explain seed survival after being digested by wild ungulates?

**DOI:** 10.1007/s00442-024-05538-7

**Published:** 2024-04-25

**Authors:** Barbora Lepková, Tereza Mašková

**Affiliations:** 1 Department of Botany, Faculty of Science, Charles University, Benátská 2, 128 43, Prague, Czech Republic; 2 Institute of Plant Sciences, Ecology and Conservation Biology, Faculty of Biology and Preclinical Medicine, University of Regensburg, 93040 Regensburg, Germany

**Keywords:** Dry grasslands, Endozoochory, Wild ungulates, Seed dispersal, Seed traits

## Abstract

Plants inhabiting open landscapes are often dispersed by ungulates and are expected to be adapted to this type of dispersal through their seed traits. To find which traits help seeds survive the passage through digestion of wild ungulates, we conducted a comprehensive feeding experiment with almost forty species of plants and three species of ungulates. We fed specified numbers of seeds to the animals, collected the dung, and germinated the dung content. We explored whether seed morphological traits and seed nutrient contents are good predictors of seed survival after passage through the ungulate digestive system. We also tested how the seed survival differed after the passage through different ungulate species. To find answers, we used GLMM with beta-binomial distribution and animal and plant species as random factor, respectively. We found that species survival and germination success were negatively correlated to seed elongation and the thickness of the seed coat. Even though phylogenetically correct GLMM did not yield significant results, when we tested species from commonly represented families, separately (legumes and grasses compared to all other species) different traits had statistically significant effects. In the case of seed elongation, the effect changed direction from negative to positive when legumes and grasses were left out. Our results suggest that seed traits enabling species survival after passage through the digestive tract are strongly phylogenetically conserved and different groups of plants evolved different ways of adapting to grazing pressure and utilize it for dispersal.

**Supplementary Information:**

The online version contains supplementary material available at 10.1007/s00442-024-05538-7.

## Introduction

Effective dispersal of plants is crucial for the connection between different habitat patches, survival of plant populations and the metapopulation dynamics, and the genetic diversity (Lozada-Gobilard et al. 2021). Large animals such as mammals, are potential drivers for both short- and long-distance dispersal (Cain et al. 1998). Two types of animal-mediated dispersal are possible: external (epizoochory, dispersal of propagules attached to the fur or hooves) and internal (endozoochory, dispersal after passage through the digestive tract). Endozoochory can be further distinguished based on the presence of fleshy fruit or other reward (mainly dispersed by frugivores) compared to species without fleshy fruits (mainly dispersed by grazing animals, e.g., ungulates) (Green et al. 2019). The effectivity of both types of dispersal as measured by the number of dispersed and germinated seeds is dependent on the characteristics of both parties: the dispersed plant species and the dispersal vector (animal). Different traits influence epizoochory while others drive endozoochorous dispersal (Albert et al. 2015). Contrary to epizoochory, where the morphology of dispersed propagule clearly affects its ability to attach and remain in fur (Albert et al. 2015; Tackenberg et al. 2006), in endozoochory by grazing animals the traits supporting successful passage through the digestive tract are less clear (Green et al. 2022). Combination of plant and animal vector characteristics affects the probability with which the seed is eaten and survives the passage through the digestive tract.

Among characteristics influencing survival in the digestive tract, seed traits are the main driver. The propagule must be able to survive rough mechanical and chemical conditions, e.g., molar mill and acidic environment in the stomach. Based on previous research, successful endozoochorously dispersed seeds are small and without appendages (to escape the molar mill) and exhibit long survival in the soil bank (which is similarly destructive as the chemical conditions in the stomach; Pakeman et al. 2002; Albert et al. 2015). Even though thick seed coat may present a protection from mechanical chewing, its effect on survival after passage has not been found (Bruun and Poschlod 2006). The effect of seed shape received contradictory support with better survival of round seeds (Mouissie et al. 2005) as well as elongated seeds (Albert et al. 2015; Cosyns et al. 2005) and no effect of seed shape whatsoever (D’hondt and Hoffmann, 2011).

However, what is rarely taken into account are the constraints of the ungulate species: how morphological constraints of the animal body influence dispersal (Illius and Gordon 1992). Generally, small animals are more likely to disperse only small seeds, whereas large-bodied animals are able to ingest both small and large seeds, but opposite was shown specifically for large ungulates (Chen and Moles 2015). The body size also influences the speed by which particles travel through the tract: large animals have a longer digestive system, and it takes longer for the particles to pass through it (Clauss et al. 2006). Hand in hand with feeding preferences go the feeding strategies and nutrient demands of ungulate species. Unlike hind-gut fermenters, ruminants are able to digest phytic acid, which is the main form of phosphorus storage (Raun et al. 1956). Furthermore, Cervids (e.g., deer) have higher phosphorus demands because they store it in their antlers (Landete-Castillejos et al. 2007) whereas other ruminants (e.g., Bovids) excrete the excess phosphorus in the dung (Sitters et al. 2014). However, when a plant invests resources to create nutrient rich seeds, it also most likely creates protection either chemical or mechanical so its resources are not wasted.

The effect of seed traits on survival in the digestive tract is usually tested on the species composition found in dung samples in comparison with available vegetation (Albert et al. 2015). This approach is used to suggest which plant traits help the species to be eaten and successfully survive the passage. But the probability of being eaten is a function of several factors including but not limited to plant frequency in the landscape (Lepková et al. 2018), seed production (Bruun and Poschlod 2006), and feeding preferences of the ungulate (Hofmann 1989). The feeding preferences are further influenced by a number of plant traits such as the nutritional value of foliage (Hejcmanová et al. 2016). When species found in dung are compared with the available vegetation, all these filters are included and direct deduction of traits enabling endozoochory is thus questionable. However, we need to study the traits influencing seed survival per se—to compare germination rates of seeds fed directly to animals in known quantities.

Due to the lack of comparison studies focusing on wild herbivores (see Table 1), in this paper, we aim to address the question of which seed traits drive the survival and germination success of species after passage through the digestive tract of three species of wild animals: red deer, sika deer, and mouflon. Specifically, we asked two main questions: (i) which seed morphological traits and nutrient contents drive the species’ ability to survive the passage through the digestive tract? We hypothesized that the best surviving species have a round shape, thick seed coat, and low amount of nutrients. (ii) Does the survival success differ between ungulate species? We hypothesized that body size has a positive effect on germination rate and thus the germination rate of plant species is the highest after passage through the digestive system of red deer and lowest after passage through the digestive system of mouflon (red deer > sika deer > mouflon). We chose thirty-eight species of plants from the landscape of dry grassland, both previously found in dung and from the same area but absent in dung (Lepková et al. 2018). A predefined number of seeds was fed to three different ungulates and their germination rate was established with a greenhouse experiment.

**Table 1:** Summary of the most relevant studies compared to our experiment

Reference	Herbivore included	No. of plant species	No. of species shared with this experiment	Length of germination experiment	
Mouissie et al. 2005	fallow deer	25	4	9 months	Greenhouse
Picard et al. 2015	roe deer, red deer, wild boar	6	1	2 months	Growth chamber
Dhond’t & Hoffman 2011	cattle	48	9	8 months	Greenhouse
Cosyns et al. 2005	sheep, cattle, donkey, horse, rabbit	19	8	6 months	Greenhouse
Bonn 2004	sheep, cattle	14	5	15 months	Garden
Pakeman & Small 2009	sheep	12	4	12 months	Greenhouse

## Materials and methods

We selected the plant species from the vegetation of dry grasslands in the Doupov Mountains in Western Bohemia where we studied endozoochorous dispersal by deer and boars before (Lepková et al. 2018, 50.295N, 13.070E). To focus only on seed survival during digestion process and avoid confounding by palatability for ungulates, we picked species based on two criteria: frequency of species in dung samples collected from the free ranging animals (> 5%) and vegetation in the Doupov Mountains (> 20%). Not all species were available from seed producers which resulted in a list of 39 species from which 23 species were previously known as dispersed in dung. One species—*Knautia arvensis*—did not germinate at all throughout this study. It was included in the feeding experiment, but it was excluded from all analyses. The analysed number of species was, therefore, thirty-eight.

All seeds were obtained from the commercial source Planta naturalis (plantanaturalis.com, Markvartice, CZ) from the harvest of 2015. Random amounts of seeds (hundreds in the case of large-seeded species, thousands in the case of small-seeded species) were weighed and counted to find out the exact number of seeds per gram. The obtained seed numbers per gram were then used to weigh the number of seeds to be fed. Plant species were divided into four groups according to their weight: extra heavy (more than 10 mg), heavy (1–10 mg), medium (0.1–1 mg) and light (less than 0.1 mg). The total number of seeds in fed mixtures reflected their absolute weight so that animals were not fed too large quantities of large and heavy seeds (for numbers of seeds fed to one individual, see Table 2). Not enough seeds were available for *Vicia tetrasperma*, and we used 400 and 200 seeds for deer and mouflon, respectively.

**Table 2:** The number of seeds fed to each animal. The numbers of individuals per animal species are in brackets. Thus, the number of seeds of a light plant species fed to all individuals of red deer was 30 000

Herbivore	Extra heavy	Heavy	Medium	Light	*V. tetrasperma*
Red deer (3) Sika deer (3)	500	1 000	5 000	10 000	400
Mouflon (7)	250	500	2 000	5 000	200

The experiment was conducted in forest ZOO Malá Chuchle in Prague, Czech Republic. Three species of ungulates were used for the experiment: three individuals of red deer (*Cervus elaphus*), three individuals of sika deer (*Cervus nippon*), and seven individuals of mouflon (*Ovis musimon*). Even though all chosen ungulate species are ruminants with a similar digestive system, both species of deer belong to the group of intermediate feeders, whereas mouflon is a more selective browser (Hofmann 1989). Different feeding behaviors lead to differences in the processing of forage in the digestive system (Baker and Hobbs 1987). See Table 3 for a summary of herbivore characteristics.

**Table 3:** Characteristics of ungulate species used in this study

Herbivore	No. of individuals	Average body size (Andera & Gaisler 2012)	Retention time	Feeding strategy
Red deer	3	60–200 kg	61 h (Müller et al. 2013)	Ruminant
Sika deer	3	28–60 kg	43 h (Asano et al. 2007)	Ruminant
Mouflon	7	20–60 kg	38 h (Behrend et al. 2004)	Ruminant

For the feeding experiment, we divided the plant species into two groups to make the identification of seedlings easier and not to feed the animals such large quantities of seeds. The groups of plant species were fed separately in two different phases: on 4-Dec-2016 and on 20-Feb-2017. All individuals of one animal species were held in an enclosure together. The seeds were fed in a mixture of oat, barley, carrot and apples with lukewarm water and all animals were fed all species of plants. The animals were fed alfalfa hay and their usual forage (fruit, dry bread, and cereals) for three days before the experiment, during the experiment, and until the end of the dung collection. All dung was removed from the paddocks before the experiment and it was used as a control sample to check for germination from dung before the feeding started. After the feeding, the dung was collected every day for four days, with few exceptions so as not to disturb the untamed animals. The period of four days was based on literature information about the retention times of all animals (Table 3). All dung present in the paddock was collected each day. All dung from one animal species and day was pooled together. The collected dung was stratified in a freezer at -18 °C for two months and then crumbled and air-dried. Using such low temperatures does not affect seed germination very much (Seglias et al. 2020).

Seed survival was checked by a greenhouse emergence experiment over a period of two and half years. The germination pots were filled with perlite, covered by non-woven fabric and the air-dried crumbled dung was put on top in 2 cm layer. This allowed the samples to be mixed without mixing the substrate as well and burying the seeds at the bottom of the pot. The emergence took place under natural light conditions from May and June 2017 for the first and second phases, respectively. Pots were watered as necessary with rain-water and shifted around the greenhouse at random. In the last months of the experiment when germination decreased, samples were left to dry out several times and mixed to let seeds from lower layers to germinate. Three different controls were set: (i) pots with dung samples collected from each enclosure before the feeding started, (ii) pots with no seeds and no samples to control for seed rain (small amount of potting soil was used on top of the non-woven fabric as substrate), and (iii) pots with 500 seeds per plant species to establish germination rate without passing through the guts (mixed with small amount of potting soil). In all cases, pots were filled with perlite and covered with non-woven fabric. All controls were stratified for the same period as the samples.

### Seed traits

We measured seed nutrient reserves as the content of nitrogen (N), phosphorus (P), carbohydrates, and oils. Carbohydrates content was calculated as the sum of fructans and starch. N content was measured by flow injection analysis after Kjeldahl mineralization. P content was measured by flow injection analysis after perchloric acid mineralization. Starch and fructans were measured with the enzymatic procedure Megazyme (McCleary et al., 1994). Oil content was measured by Soxhlet extractor (International Organization for Standartization 2016). Several grams per species were used for the chemical analyses, see Mašková and Herben (2021) for more details. We measured seed coat thickness on dissected seeds under a light microscope. Four measurements on five seeds were taken and species-specific mean value of seed coat thickness was calculated. To calculate the index of shape (Thompson et al. 1993) we used seed dimensions from LEDA database (Kleyer et al. 2008). The index is the variance in dimensions and it may range from 0 (perfectly round seeds) to 0.22 (extremely elongated seeds). The following equation was used to calculate the index:

$$\frac{\sum {\left({x}_{i}-\underline{x}\right)}^{2}}{n},$$

where x_i_ represents seed dimension (length, width or height), x represents mean of seed dimensions, and n represents number of seed dimensions (3 in our case).

To make the understanding of the index simpler, it is further referred to as seed elongation.

### Data analyses

No plant species germinated better after passing through the digestive system than the unpassed controls. We thus used the germination rate of controls as the maximum possible germination potential and corrected the number of fed seeds for this germination potential. Pearson correlation coefficient was calculated for each pair of tested variables before building models. However, due to high variation in variables with correlates and important information each variables holds separately, no variable was omitted from the analyses.

We used a generalized linear mixed-effect model (GLMM) to test the effect of seed traits on species germination rate. We used package glmmTMB (Brooks et al. 2017) with beta-binomial distribution to account for overdispersion and included animal species as a random factor. To correct for plant phylogeny in the effect of seed traits on plant germination we used package phyloglmm (Li and Bolker 2023). Similarly, we used GLMM with betabinomial distribution to test for the differences between animal species (plant species as random factor). For the tests with betabinomial distribution, we made a matrix of successes (germinated seeds) and failures (germinated seeds subtracted from the fed seeds). Furthermore, we separated species into three major groups and tested the effect of plant traits on these species separately: Fabaceae (9 species), Poaceae (14 species), and all other species (15). Models for separate plant groups were made in the same manner (GLMM with betabinomial distribution and animal species as random factor).

We used the RLQ method (Kleyer et al. 2012) from the package ade4 (Thioulouse and Dray 2007) to test the correlation between seed traits, seed germination rate, and animal species. These three types of information were all stored separately in three matrices. The RLQ analysis consisted of several steps: first, individual matrices were analysed using principal component analysis (seed trait matrix and matrix of “environmental” variables, here the species of animal) and correspondence analysis (species germination rate matrix). The final RLQ model was then tested using fourth-corner statistics (function forthcorner.rlq, package ade4).

All analyses were performed in R (version 4.3.1., R Core Team 2023). Nomenclature of plant species follows Kubát et al. (2002).

## Results

**Fig. 1:**
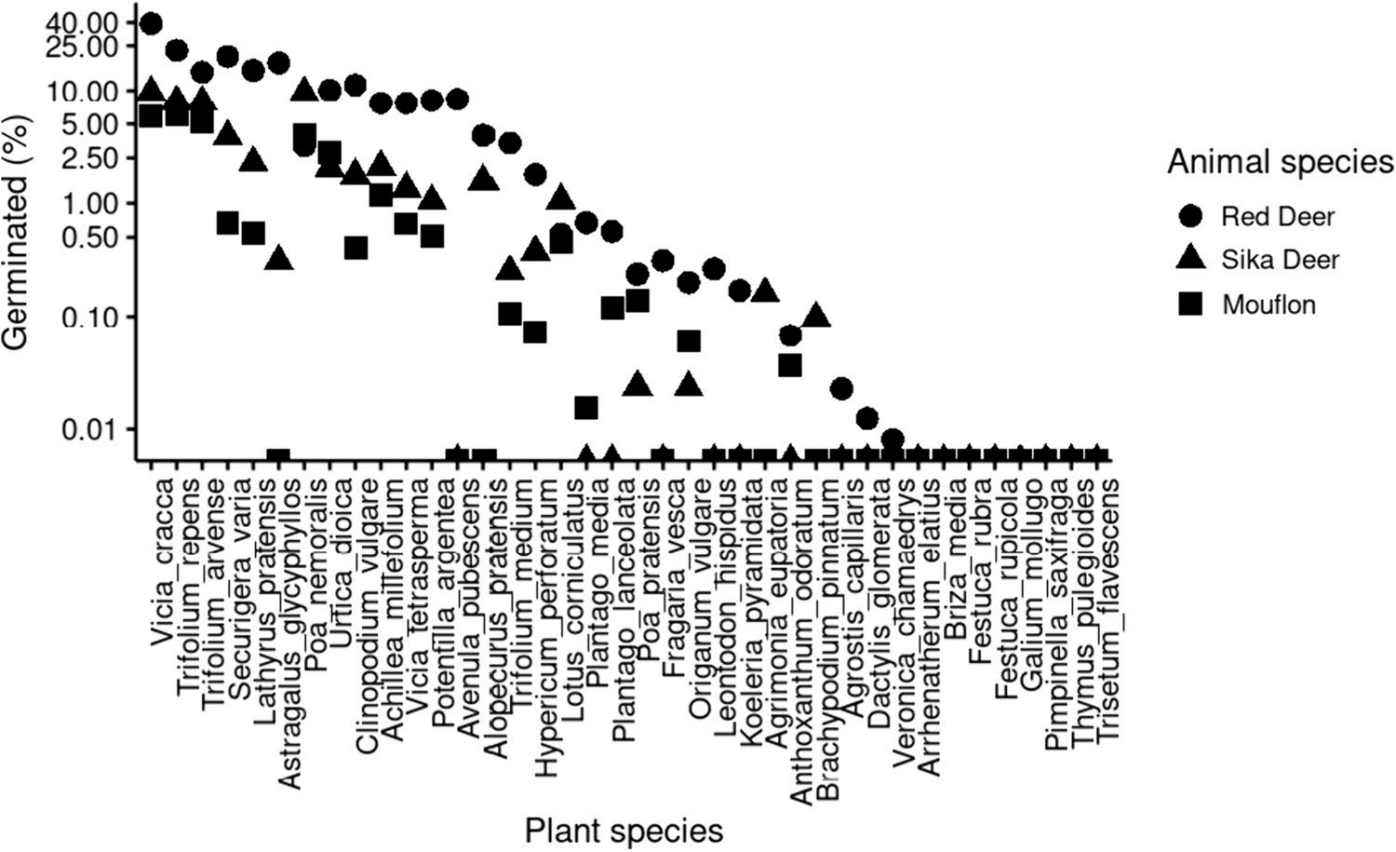
The seed germination rate after passage through herbivore guts and how the survival differs between herbivore species: red deer (full circle), sika deer (full triangle), and mouflon (full square). *Vicia cracca* was the best surviving plant species, with germination after passage through red deer nearing 40%. Species with zero germination after passage through all three herbivore species were not plotted. The survival rate is depicted as a percentage of the number of seeds ingested corrected for the non-ingested seeds

None of the studied species showed increased germination after passage through the digestive tract, and eight species did not germinate from dung samples at all. The germination rate ranged from 0 to 39% (percentage of germinated seedlings from the total number of fed seeds corrected for germination rate of seeds in the control lot). The most successful species belonged to the Fabaceae family: *Vicia cracca*, *Trifolium repens*, *Securigera varia*. However, even in the species with the highest germination rate, it varied between animals: the best surviving *Vicia cracca* showed a germination rate of almost 40% after passage through red deer but less than 6% after passage through mouflon. Plant species survived the passage through red deer better than through other herbivores (Fig. 1.).

### Seed traits and nutrient contents

The effect of seed traits on germination rate was tested both with and without phylogeny. Accounting for phylogeny resulted in a non-significant effect of any of the tested variables, however, the germination rate differed significantly between three tested groups of plant species: legumes (Fabaceae), grasses (Poaceae), and other species. Without accounting for phylogeny, the germination rate of all plant species after passage through digestive tract was negatively affected by seed elongation and by the thickness of seed coat (see Table 4, first column). In all variables, the plant species with zero or extremely low germination rates were distributed along the entire measured gradient but no model with zero inflation argument improved the fit.

**Table 4:** Results of the GLMM of all species, only Fabaceae, only Poaceae, and all other species. P values for the significant variables are in bold. The effect direction (positive or negative) is indicated with the direction of the Estimate. A different trend is visible for the seed elongation with a negative effect in Fabaceae, positive in other species, and non-significant in Poaceae

	All Species		Fabaceae		Poaceae		Other Species	
variable	Estimate	p-value	Estimate	p-value	Estimate	p-value	Estimate	p-value
Seedmass	0.718	0.054	0.784	0.389	-15.145	0.141	2.752	0.201
P	-0.040	0.972	3.447	**0.014**	-7.015	0.129	33.994	**0.001**
N	0.248	0.083	-1.561	**0.002**	-0.131	0.913	-6.660	**0.001**
Starch	-0.019	0.292	-0.178	**0.037**	0.104	0.066	-0.599	0.494
Oil	-0.043	0.061	-0.046	0.856	0.105	0.433	-0.115	0.104
Elongation	-13.645	**0.003**	-29.198	**0.021**	-9.783	0.759	37.763	**0.001**
Seed coat thickness	-6.695	**0.038**	2.933	0.890	37.991	0.051	-3.502	0.763

**Fig. 2:**
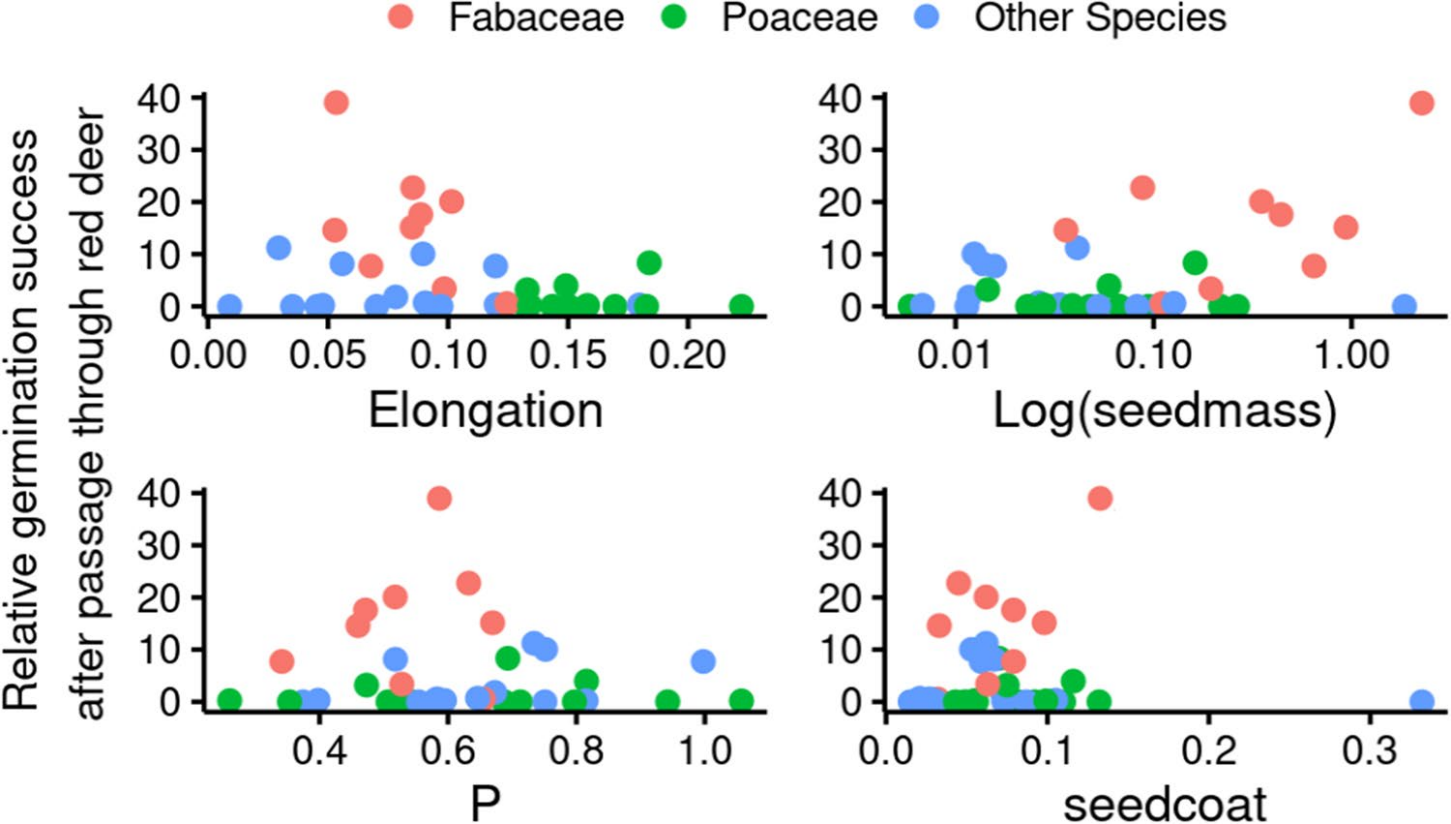
The relationship between significant seed traits and nutrient values, and the relative germination success for passage through red deer. The plant species were divided into three groups based on their family: Fabaceae species in pink, Poaceae species in green, and all species from other families in blue (Other Species). Seed mass is in log scale

The trait analyses have also been done separately for the families with the highest number of species: Fabaceae and Poaceae, and for the remaining species. Different results were found for individual groups of species. In some cases, we found a positive effect of a trait or nutrient in one group and a negative in another (Table 4, Fig. 2 for red deer). For example, the effect of seed elongation was negative (better survival of round seeds) in Fabaceae but in the group of other species it had a positive effect (better survival of elongated seeds). In the case of seed phosphorus content, it had a significantly positive effect in Fabaceae and in the group of other species, but it became non-significant in the test for all species. On the other hand, the seed coat thickness had a significantly negative effect in the test of all species but became non-significant in all of the separate tests.

### Differences between ungulates

**Fig. 3:**
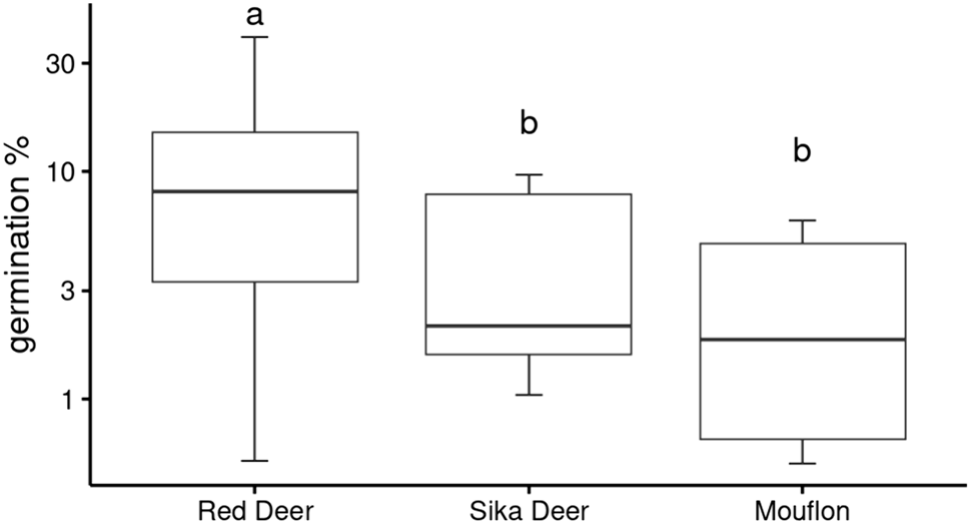
The germination rate of seeds after passage through three tested species of herbivore: red deer, sika deer, and mouflon. The letters above boxplots show the significant differences between animals as tested by a mixed-effect model with a beta-binomial family where the plant species was set as a random factor. The only significant difference was in red deer, which showed higher germination success than all other animals

There was no significant relationship between seed traits and the species survival after passage through different species of animals as tested by RLQ and fourth corner analysis. However, there were significant differences in the survival of seeds after passage through different species of ungulates (GLMM, p < 0.001). Post hoc tests revealed that after passage through red deer germination was significantly higher than after passage through any other tested ungulate (Table 5, Fig. 3).

**Table 5:** Results of the GLMM testing the effect of herbivore species on the germination success of ingested seeds of 38 plant species. Post hoc pairwise comparison was performed using package emmeans and revealed that survival of seeds after passage through red deer differed from all other animal species. The remaining animal species did not differ from each other

contrast	estimate	SE	df	p-value
Mouflon—Red Deer	-1.930	0.216	146	** < .001**
Mouflon—Sika Deer	-0.541	0.232	146	0.051
Red Deer—Sika Deer	1.390	0.180	146	** < .001**

Interestingly, red deer dung samples collected after four days from the feeding trials still contained seeds of some species suggesting the retention time was underestimated. This was the case for *Alopecurus pratensis*, *Trifolium arvense*, and *Clinopodium vulgare*, and at a lower rate for *Hypericum perforatum*, *Plantago media*, *Potentilla argentea*, and *Securigera varia* (Fig. 1). However, this has not been the case for other animal species, even though in sika deer, the collection was omitted on day 3 in phase 2.

## Discussion

None of the species showed increased germination after passing through the digestive tract. The effect of seed traits was in majority contrary to our predictions and in some cases, we found contrasting results when groups of plant species were tested separately. For example, the seed elongation (index of shape) had negative effect in Fabaceae but positive in the group of other species. Contrasting results can be found in the literature: better survival of round seeds was reported by Mouissie et al. (2005), better survival of elongated seeds in Cosyns et al. (2005), and no significant relationship between seed survival and shape in D’hondt and Hoffmann (2011). The cited studies used a range of plant species but only in the last case did the authors take phylogenetic relationships into account. Even though seed traits are often phylogenetically conserved (Moles et al. 2005), analyses with phylogeny might not receive significant effects (Bello et al. 2015), as was also our case. This suggests that both the tested traits and plant adaptation to the environmental filter (here, the passage through herbivore guts) are phylogenetically conserved (Bello et al. 2015). Furthermore, in the cited studies (Cosyns et al. 2005; Mouissie et al. 2005; D’hondt and Hoffmann, 2011), the seed shape measurements used were made on seeds with no appendages, whereas in the presented study we used data from the LEDA database where seeds were measured with appendages. When grazing, ungulates feed on vegetation and the seeds they consume are not cleaned of glumes or pericarps. However, such structures can provide extra protection for seeds in the digestive tract, and therefore, they should be included in the measurements. The question remains when during the digestive passage do the appendages fall off. The appendages may fall off during the chewing and mastication, thus providing mechanical protection. It is possible the appendages stay on the seeds until they reach the stomachs and fall off during the digestive process per se, thus providing chemical protection. Unfortunately, such data are unavailable. Furthermore, there is an evolutionary difference between the groups of species we tested separately and our results suggest that individual groups of species adapted differently to the grazing pressure.

Throughout the studied species set we found surprisingly low germination rate of plant species frequently found in dung samples, e.g., *Poa pratensis* or *Veronica chamaedrys*. Two reasons are plausible: (i) seeds from the commercial supplier were of insufficient quality. This is not probable because the species showed successful germination in the control pots. (ii) The animals in the field ingest numbers of seeds several orders of magnitude higher than what we fed them in the experiment. This suggests preferential grazing which is not related to seed traits or their nutritional content but can be driven by other traits of the mother plants which influence palatability.

Previous research showed that even a very similar setting of feeding experiment does not guarantee similar outputs. For example, in a multi-species study with a number of plant species fed to cattle, Cosyns et al. (2005) and D’hondt and Hoffmann (2011) found vastly different results in the species of plants included in both studies, e.g., the relative germination rate of *Agrostis capillaris* was 17 and 54%, respectively. In the presented study, the measured germination rate for the same plant species was effectively zero, no matter the ungulate species, even though the germination rate of control seeds was 73%. However, this species is one of the most common grasses dispersed by wild ungulates (present in 35% and 20% of deer and wild boar dung samples collected directly in the field, respectively; Lepková et al. 2018). This shows high discrepancy between different types of experiments. Even similar experiments give different results under the influence of naturally behaving animals. Seed dispersal is under the direct influence of animal personality (Zwolak and Sih 2020). Furthermore, the digestion changes under the effect of other forage the animal consumed together with the tested seeds (Ramanzin et al. 1997), it differs between males and females and changes between seasons (Zhu et al. 2020).

Species from the Fabaceae family showed the highest germination rate after passage through the guts (similarly to Gardener et al. 1993a). However, results of laboratory experiments (Milotić and Hoffmann 2016), can be vastly different which suggests such processes need to be tested with real animals. The success of the Fabaceae family is often explained by their mechanical characteristics (e.g., thick seed coat; Gardener et al. 1993a). In the case of the presented study, the best surviving Fabaceae had round seeds with high amount of phosphorus and low amount of nitrogen and starch but the thickness of seed coat did not have a significant effect.

Our experiment revealed a complex relationship between seed survival in the guts and the content of available nutrients. Only starch had significant effect when all species were tested together. Surprisingly, no nutrient had significant effect on the germination rate of Poaceae (Table 4). Both legumes and grasses are known for specific amounts of seed nutrients (Mašková and Herben 2021), and we expected these nutrient contents to affect the seed survival. However, we revealed relationships largely contrary to our predictions. In Fabaceae and the group of other species (legumes and grasses excluded), we found a significant positive effect of phosphorus content on seed survival even though ruminant herbivores are preadapted to digest the phytic acid in which phosphorus is stored (Klopfenstein et al. 2002). The effect of phosphorus on the survival of grasses was non-significant and, similarly, the overall test of all species did not show a statistically significant effect. Furthermore, the individual groups of species, Fabaceae, Poaceae and the other species, underwent different evolutionary paths. This suggests species from the groups in question are evolutionary preadapted to consumption and possible dispersal differently.

### Differences between ungulate species

Three ungulates exhibiting different feeding and digestive behavior were used for the experiment, and we expected differences based on their body size. Our results support our hypothesis that seed survival is higher after passage through larger animal compared to smaller ones even though we did not find any specific traits supporting seed survival after passage through different species of animal via the RLQ analysis. Dung samples from red deer, as the largest animal (Anděra and Horáček 2005, Table 3), showed the highest germination rates of seedlings and it differed significantly from the other ungulates (Table 3). The difference between sika deer and mouflon were on the verge of significance, and these animals have very similar body size (Table 3). However, the effect of body size is contradictory in other literature using feeding experiments. Simao Neto et al. (1987) recorded the highest germination rate after digestion by cattle followed by goats and sheep. On the other hand, Cosyns et al. (2005) reported the highest germination rate after digestion by donkeys and rabbits compared to cattle, horses and sheep and stated no simple relationship between animal digestive system or body weight and germination rate of digested seeds. Studies comparing multiple animal species are rare (Cosyns et al. 2005) and other factors may play role, e.g., the domestication status. Furthermore, both studies use much lower number of plant species (6 in Simao Neto et al. 1987 and 19 in Cosyns et al. 2005) and the species selection may have a strong influence on the reported results. Chen and Moles (2015) performed a meta-analysis on the relationship between seed size, seed dispersal, and disperser body size. They found out that specifically among large ungulates the relationship with seed size is negative, i.e., the large animals primarily ingest small-seeded species. The relationship is even more complicated because ruminants spit large seeds which are not digested at all (Castañeda et al. 2018) but this was not the case of presented experiment as we worked with relatively small-seeded species and never observed spitting.

### Speed of passage

Since some species were still germinating in significant numbers from samples from the fourth day of collection, we must assume the retention time was longer than 96 h (but see Cosyns et al. 2005 and Table 3). This was particularly true in the case of red deer where the germination rate of ten plant species from samples collected on day 4 was higher than from samples collected on day 3. However, in only one plant species the difference was substantial (*Alopecurus pratensis*, 50-fold increase). In general, Fabaceae had a slower passage through the guts (similar to Gardener et al. 1993b) and also the highest measured survival success. This is in contrast to our prediction that long passage through the digestive tract shall be more destructive for the seeds. However, this result also means that seeds, which stay in the digestive tract for this long, can be dispersed further away from the mother plant which can even compensate for losses during the passage (Janzen 1984).

## Conclusions

Our experiment with almost forty species of plants and three species of ungulates did not reveal a single seed trait that predicts seed survival when passing through the ungulate digestive system. The most important traits were seed mass and seed coat thickness but when we analyzed plant species from different families individually, other traits drove the seed survival, particularly the seed nutrient content and seed shape. Our results suggest that plant families under strong grazing pressure, i.e., Fabaceae and Poaceae, followed a different evolutionary path. Based on our comprehensive experiment, we conclude, that digestion by wild, free-ranging ungulates does not act as environmental filter. Seed survival after passage through the digestive system is driven by seed traits according to different evolutionary trajectories of different plant families. Disperser characteristics are important to take into account as well, e.g., the body size.

### Supplementary Information

Below is the link to the electronic supplementary material.

Supplementary file1 (XLS 49 KB)

## Data Availability

Not applicable.

## Availability of data and material

All data produced from this study are provided in this manuscript.
